# Endoscopic membrane resection using a scissors-type knife in a pediatric patient with congenital duodenal membranous stenosis

**DOI:** 10.1055/a-2208-6728

**Published:** 2024-01-09

**Authors:** Jun Nakamura, Takuto Hikichi, Hideaki Tanaka, Minami Hashimoto, Tsunetaka Kato, Takumi Yanagita, Hiromasa Ohira

**Affiliations:** 1 215686 Endoscopy, Fukushima Medical University Hospital, Fukushima, Japan; 2 183174 Gastroenterology, Fukushima Medical University School of Medicine, Fukushima, Japan; 3 215686 Pediatric Surgery, Fukushima Medical University Hospital, Fukushima, Japan

Congenital duodenal membranous stenosis (DMS) is a narrowing of the lumen caused by formation by the mucosa of membrane-like structures; it is a rare disease with a frequency of 1 in 10.000–40.000 1 . Notably, some endoscopic treatments for congenital DMS have been reported 1 2 3 4 ; however, the endoscope’s maneuverability in pediatric patients’ duodenums is limited because of their small physique. Herein, we present a case of congenital DMS in which a scissors-type knife was successfully used to treat the membranous stenosis.

A 5-year-old boy with frequent vomiting underwent esophagogastroduodenoscopy (EGD) under general anesthesia. EGD and duodenography with a contrast agent revealed DMS in the descending part of the duodenum, and the papilla of Vater was observed on the anal side of the DMS ( Fig. 1 ).

**Fig. 1:**
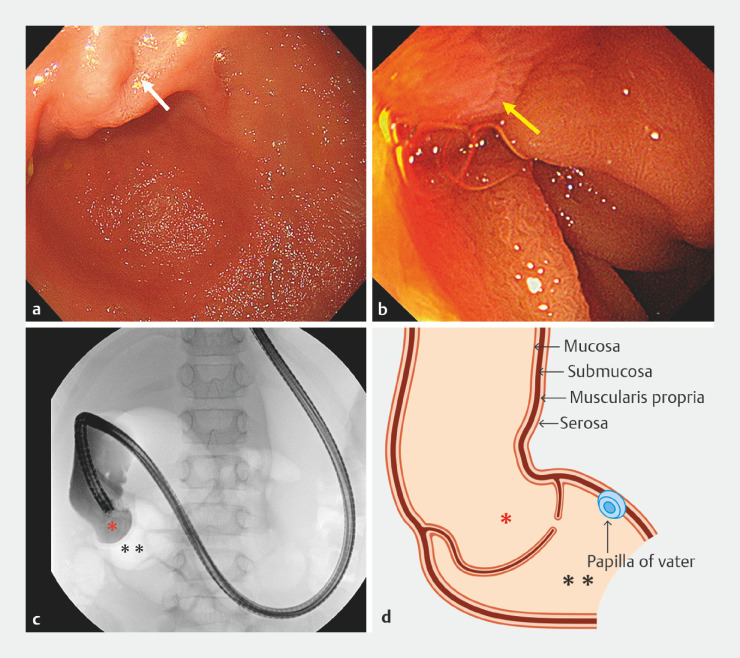
The pre-treatment appearance on: **a** endoscopy, showing a membranous stenosis in the descending part of the duodenum (white arrow), with dilatation on its oral side (the duodenum on the anal side could not be observed using a conventional scope); **b** endoscopy using an ultrathin endoscope, showing the papilla of Vater (yellow arrow) on the anal side of the membranous stenosis; **c** duodenography, showing contrast retained above the membranous stenosis (red asterisk), with no flow to the anal side (black asterisks) owing to obstruction in the descending portion of the duodenum; **d** a schema, showing the congenital duodenal membranous stenosis separating the oral (red asterisk) and anal lumens (black asterisks).

Endoscopic treatment was performed after informed consent had been obtained ( Video 1 ). A guidewire was placed over the DMS as a landmark for the lumen ( Fig. 2 **a** ). First, an incision was made using an ITknife nano (Olympus Co., Tokyo, Japan) with a therapeutic endoscope, while avoiding the papilla of Vater ( Fig. 2 **b** ). However, the patientʼs narrow duodenum limited the working space, making it difficult to move the endoscope precisely. Therefore, we switched to performing the procedure using a scissors-type knife (SB Knife Jr2; SB-Kawasumi, Kanagawa, Japan), which is able to rotate without moving the endoscope ( Fig. 2 **c** ). As a result, the DMS was successfully incised, and the endoscope could be passed easily through the lumen ( Fig. 2 **d** ). The patient was asymptomatic 2 months after the procedure.

Congenital duodenal membranous stenosis in a pediatric patient is treated with an endoscopic procedure using a scissors-type knife.Video 1

**Fig. 2:**
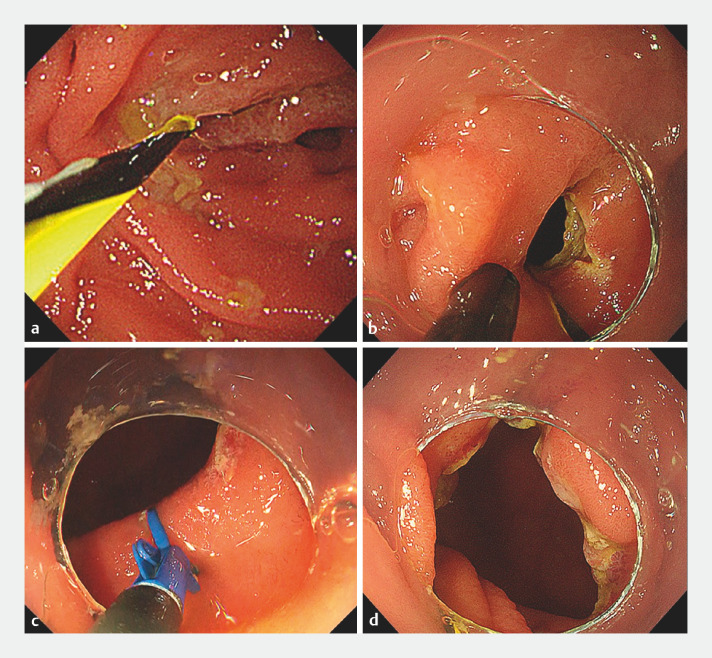
Endoscopic images during the treatment showing: **a** a 0.025-inch guidewire passed beyond the membranous stenosis to identify the position of the lumen; **b** the initial incision of the duodenal membranous stenosis made using an ITknife nano, with poor endoscopic maneuverability making continuation difficult; **c** the scissors-type knife, which can be rotated without moving the endoscope, being used to continue the incision; **d** the widened lumen after endoscopic treatment, through which the endoscope could easily be passed.

To our knowledge, no previous reports of the treatment of congenital DMS using scissors-type knives exist. An endoscopic procedure using a scissors-type knife is a safe and effective treatment option for congenital DMS, even where endoscopic manipulation in the duodenum is difficult.

Endoscopy_UCTN_Code_TTT_1AQ_2AZ

## Notes

Endoscopy E-Videos https://eref.thieme.de/e-videos
*E-Videos* is an open access online section of the journal *Endoscopy* , reporting on interesting cases and new techniques in gastroenterological endoscopy. All papers include a high-quality video and are published with a Creative Commons CC-BY license. Endoscopy E-Videos qualify for HINARI discounts and waivers and eligibility is automatically checked during the submission process. We grant 100% waivers to articles whose corresponding authors are based in Group A countries and 50% waivers to those who are based in Group B countries as classified by Research4Life (see: https://www.research4life.org/access/eligibility/ ). This section has its own submission website at https://mc.manuscriptcentral.com/e-videos .
